# Ecologically relevant biomarkers reveal that chronic effects of nitrate depend on sex and life stage in the invasive fish *Gambusia holbrooki*

**DOI:** 10.1371/journal.pone.0211389

**Published:** 2019-01-28

**Authors:** Oriol Cano-Rocabayera, Adolfo de Sostoa, Francesc Padrós, Lorena Cárdenas, Alberto Maceda-Veiga

**Affiliations:** 1 Department of Evolutionary biology, Ecology and Environmental Sciences–Institute of Research in Biodiversity (IRBio-UB), Universitat de Barcelona, Spain; 2 Servei de Diagnòstic Patològic en Peixos, Facultat de Veterinària, Universitat Autònoma de Barcelona, Bellaterra, Spain; VIT University, INDIA

## Abstract

Agricultural intensification and shifts in precipitation regimes due to global climate change are expected to increase nutrient concentrations in aquatic ecosystems. However, the direct effects of nutrients widely present in wastewaters, such as nitrate, are poorly studied. Here, we use multiple indicators of fish health to experimentally test the effects of three ecologically relevant nitrate concentrations (<10, 50 and 250 mg NO_3_^-^/l) on wild-collected mosquitofish (*Gambusia holbrooki*), a species widely introduced for mosquito biocontrol in often eutrophic waters. Overall, biomarkers (histopathology, feeding assays, growth and caloric content and stable isotopes as indicators of energy content) did not detect overt signs of serious disease in juveniles, males or females of mosquitofish. However, males reduced food intake at the highest nitrate concentration compared to the controls and females. Similarly, juveniles reduced energy reserves without significant changes in growth or food intake. Calorimetry was positively associated with the number of perivisceral fat cells in juveniles, and the growth rate of females was negatively associated with δ^15^N signature in muscle. This study shows that females are more tolerant to nitrate than males and juveniles and illustrates the advantages of combing short- and long-term biomarkers in environmental risk assessment, including when testing for the adequacy of legal thresholds for pollutants.

## Introduction

Nutrient pollution results in man-made eutrophication, which is amongst the most pernicious forms of global change affecting aquatic ecosystems around the world [1,2]. Human causes of eutrophication are the inefficient use of fertilizers, aquaculture and urban outflows and atmospheric nitrogen deposition from combustion [3–5]. The ecological effects of eutrophication are well-known, including toxic algal blooms and high mortality of animals due to dissolved oxygen depletion at night [6–8]. Water authorities attempt to mitigate eutrophication by establishing safe nutrient concentrations (e.g. OECD, 1982; Directive 91/676/ECC [9,10]). However, the direct toxicity of nutrients to wildlife under chronic exposure is still poorly studied [11,12]. Considering agricultural intensification continues unabated and water purification is costly [13], there is the pressing need to get better insight into the health effects that environmentally relevant nutrient concentrations have on wildlife.

Nitrate (NO_3_^-^) is a widely distributed nutrient that naturally occurs at a low environmental concentration [3]. However, it can reach up to 2000 mg NO_3_^-^/l in aquaculture tanks and 345 mg NO_3_^-^/l in surface waters in nitrate vulnerable zones [14,15]. From 2012 to 2015 the surface area vulnerable to nitrate pollution increased from 1951898 km^2^ to 2175861 km^2^ just in Europe, representing 61% of the total agricultural area [16]. Alongside surface waters, nitrate pollution degrades groundwater, with reported concentrations of more than 395 mg NO_3_^-^/l [17], which exceeds the legal thresholds for Europe (50 mg NO_3_^-^/l; Directive 91/676/ECC [10]) and U.S. (44 mg NO_3_^-^/l; USEPA SWDA [18]). Ground and surface waters are linked, buffering groundwater against shortages of surface water during drought [7]. Moreover, climate change may intensify the effects of nitrate pollution on temperate rivers if shifts in precipitation regimes increase agricultural run-off [2].

Nitrate toxicity has long attracted the attention of public health agencies after nitrate-induced oxidation of respiratory pigment (methemoglobinemia) was recorded in U.S. babies [19]. Studies have since reported diseases other than respiratory issues in humans and in laboratory and domestic animals after drinking nitrate-polluted water, including mortality, oxidative stress, hypertension, birth defects, diabetes, impaired thyroid function, spontaneous abortions or cancer [11,20,21]. For water-breathing animals, nitrate was generally considered of little concern, possibly because nitrate has low branchial permeability compared to the highly toxic ammonia and nitrite [22,23]. This view changed after experimental evidence showed methemoglobinemia and alterations in hormone levels, behaviour, growth or in vulnerability to diseases in aquatic taxa under chronic nitrate exposure [11,24,25]. However, these studies used eggs, juveniles or adults of one sex of different species, all of which are factors that may affect the toxic response [26]. Moreover, toxic responses can be delayed, so that the combined use of short- (e.g. feeding assays) and long-term biomarkers (e.g. growth) will provide a more holistic view of nitrate toxicity to wildlife than the often used single-type biomarker approach [27].

The eastern mosquitofish (*Gambusia holbrooki*) is one of the world’s worst piscine invaders, which has been introduced in many temperate regions due to a misguided strategy for mosquito control [28]. Although extensively used in ecotoxicology, nitrate toxicity to mosquitofish has not been examined in detail. Reduced sperm counts and increased testicular weight in male mosquitofish were associated with concentrations of up to 22 mg NO_3_^-^/l in U.S. streams [29]. However, there is no experimental evidence for other nitrate-induced alterations in mosquitofish. The effects of nitrate on other fish species are negative [11,24,30], almost neutral [31] and even protective against a disease [32]. Nevertheless, these studies were mostly conducted on captive-reared species, which may have a higher tolerance to nitrate than wild fish because nitrate accumulates in aquaculture tanks and nitrate pre-exposure increases tolerance [33]. This rationale may apply to wild taxa if pre-exposure to the many pollutants occurring in natural waters induces co-tolerance [34].

The present experimental study monitored over 8 weeks the effects of three ecologically relevant nitrate concentrations on wild males, females and juveniles of mosquitofish using endpoints associated with their ecological impact. If males are the sicker sex [35,36,37] and juveniles are more vulnerable than adults to pollution [26,38], then we expected female mosquitofish to be the most tolerant to nitrate pollution. If the effects of nitrate pollution are subtle, then we expected nitrate effects to be more apparent in short- than in long-term biomarkers. Finally, responses in nitrate treatments should be comparable to those in controls if mosquitofish can cope with nitrate toxicity. Given the ecological relevance of the biomarkers used, our work will explore whether the ecological impact of the mosquitofish can be modulated by changing water-nutrient concentrations.

## Materials and methods

### Fish origin and general fish maintenance

The male and female mosquitofish used in this study were captured with dip nets in November 2012 in channels draining an agricultural area in the Llobregat river, Barcelona, Spain (41°16’52”N, 2°02’04”E). Fish were brought to the University of Barcelona in opaque plastic tanks provided with air-pumps and were acclimatised for one week to the laboratory conditions in two mixed sex stock 500 L tanks provided with an external filter, artificial plants and flowerpots for refugee. Fish were maintained in acclimation and experimental conditions as follows. A malaquite green/formaline bath was applied at a prophylactic dose upon arrival (see [39]). Water was then fully renewed by using dechlorinated tap water as we did to maintain the experimental environmental conditions (see section 2.3). Water properties in the laboratory tap were: pH = 7.7, mg/l, ammonia <0.5 mg/l, nitrite <0.03 mg/l, nitrate = 7.4 mg/l, sulphates = 81.2 mg/l, chloride = 130 mg/l, bicarbonate = 221 mg/l and conductivity = 784 μS/cm. Pregnant females (*N* = 15) with overt signs of giving birth soon were introduced in batches of three in 100 L tanks provided with nets to collect recently newborn juveniles (N = 165). Both adults and newborn were kept under 22±1°C and 12 h light:12 h dark cycle and fed daily with crushed commercial Sera Vipan flakes and weekly with frozen bloodworms for adults and live *Artemia* nauplii for newborns. Fish were fed once daily until satiety and uneaten food and faeces were removed daily with a dipnet. Each tank had a biological filter to prevent metabolic waste built-up (NH_4_^+^ and NO_2_^-^) and ensure water oxygenation.

### Ethics statement

The experimental procedure was authorised by the Natural Environment and Biodiversity Division at the Catalan Department of Agriculture and Fisheries (Num. DAAM 8290). Fish capture and maintenance were approved by the Committee for an Ethical use of Experimental Animals at the University of Barcelona (Num. 87/15). All fish were humanely euthanized on the termination of the experiment in compliance with Spanish legislation for the management of invasive species (Real Decreto 1628/2011 [40]).

### Experimental nitrate concentrations and exposure conditions

Sodium nitrate (NaNO_3_, CAS Number: 7631-99-4) was used to make two nitrate solutions (50 and 250 mg NO_3_^-^/l, equivalent to 11.5 and 57 mg NO_3_^—^N/l, respectively) using dechlorinated tap water, which was also used in the control treatment (<10 mg NO_3_^-^/l). The lowest nitrate concentration is the safety nitrate threshold for European waters (Directive 91/676/ECC [10]) and the highest level is within the range reported in aquaculture [24] and in rivers draining nitrate vulnerable zones in Europe [41] and tropical countries [42]. Experimental nitrate concentrations represented a 0, 5- and 25-fold increase, respectively, for mosquitofish in relation to the nitrate concentration at the collection site (9.9 ± 3.0 mg NO_3_^-^/l, based on quarterly water analyses over one year).

Male and female mosquitofish were visually size-matched per sex (total length, female: TL = 37.6 ± 0.4 and male: 25.9 ± 0.2 mm) and exposed for 8 weeks to the experimental nitrate solutions in 20 L aquaria (N = 5 tanks per treatment and sex) with six males or females in each replicate. For juveniles, the same experimental setting was used but these were exposed in batches of 11 siblings per tank. The exposure started by increasing nitrate concentrations in each aquarium drop by drop (~3 h) via a 5 mm ø tube connected to a tank with clean water or one of the two nitrate solutions. This drop-by-drop system was used to refill each tank with fresh water from each experimental condition after 50% of water was changed every three days. Water samples randomly analysed from the different treatments using the colorimetric kit VISOCOLOR indicated that the water quality conditions remained constant through the experiment at 24 h of the next water change (Table 1).

**Table 1:** Water quality properties measured in the experimental tanks.

	Control	50 mg NO_3_^-^/l	250 mg NO_3_^-^/l
Temperature (ºC)	22.5 ± 0.7	22.4 ± 0.7	22.5 ± 0.8
pH	7.8 ± 0.2	7.8 ± 0.2	7.8 ± 0.2
Carbonate hardness (KH)	9.2 ± 0.9	8.9 ± 1.0	9.3 ± 0.9
Total hardness (GH)	12.3 ± 0.8	11.9 ± 0.6	11.8 ± 1.0
Dissolved O_2_ (mg/l)	8.73 ± 0.17	8.78 ± 0.16	8.71 ± 0.16
Dissolved O_2_ (%)	98.3 ± 1.5	98.8 ± 1.1	98.1 ± 0.7
Ammonium (mg NH_4_^+^ /l)	0.08 ± 0.03	0.03 ± 0.02	0.08 ± 0.03
Nitrite (mg NO_2_^-^/l)	0.23 ± 0.07	0.26 ± 0.06	0.33 ± 0.08
Nitrate (mg NO_3_^-^/l)	14 ± 2	47 ± 3	216 ± 11
Phosphate (mg P-PO_4_/l)	0.3 ± 0.1	0.2 ± 0.1	0.3 ± 0.2

Values represent mean (±Standard Error). Data from aquaria with males, females and juveniles of mosquitofish (*Gambusia holbrooki*) combined.

### Overview of the biomarkers measured to appraise nitrate toxicity

The effects of chronic nitrate exposure on males, females and juveniles of mosquitofish were examined using 13 variables (Table 2). All of them are indicators of fish health, but calorimetry and stable isotope signatures inform the quality of fish for piscivores (see section 2.4.2). Alterations in the quantity of food eaten or in the reaction time to a stimulus show how nitrate may alter mosquitofish performance in ecosystems. Indicators related to fish growth, body condition based on mass-length relationships, energetic content and histopathology were recorded on the termination of the experiment (8 weeks). However, the feeding behaviour of mosquitofish was monitored at 0, 4, 6 and 8 weeks. All fish were euthanized at 8 weeks using an overdose of the anaesthetic MS-222.

**Table 2:** List of the biomarkers used to assess the effects of nitrate on mosquitofish (*Gambusia holbrooki*).

Biomarker	Rationale	Sample size
**Histopathology** Liver, gill	Liver is involved in the detoxification of many toxicants and these mostly entry through the gills, including nitrate. Medium to long-term response.	15M / 15F / 15J per treatment
**Calorimetry** * Energy density (J/g)	An overall measure of energy content based on the amount of heat released from the combustion of mostly lipids and glycogen. Medium to long-term response.	15M / 15F / 40J per treatment
**Stable isotopes** δ^13^C, δ^15^N, C/N	A measure of tissue composition mostly based on the effect that a change in lipid content has on the C/N ratio. Medium to long-term response.	15M / 15F per treatment
**Mass–length measures** Scaled Mass Index (SMI) Specific growth rate	Mass-length relationships, such as the SMI, indicate changes in the weight of an individual in relation to another of the same length. Specific growth rate informs the success of an individual in energy allocation to cope with pollution. Long-term response biomarkers.	30M / 30F / 55J per treatment
**Feeding behaviour** Feeding latency Voracity, Satiety	Alterations in food intake often occur in fish under stress, such as pollution exposure. Short-term response; over the long term it can translate to effects on fish survival and ecological networks.	30M / 30F / 55J per treatment

Values indicate the sample size (*N*) of male (M), female (F) and juveniles (J). Since histopatology, stable isotopes and calorimetry biomarkers implied destructive sampling; their sample size was half of the non destructive analyses.

*Males and juveniles were pooled within aquarium to meet the equipment minimum mass requirements. For juveniles, this did not allow us to use them for stable isotope analysis.

#### Growth and body condition based on mass-length relationships

Male and female mosquitofish were anesthetised with MS-222 (0.02%), measured (TL, mm) and weighted (0.001g) at Time 0 and at 8 weeks. Newborns other than those used in the experiment were used to estimate the size of experiment juveniles at Time 0 (TL = 8.9 ± 1.2 mm) to avoid compromising the health of the tiny tested individuals due to handling.

Fish size measures were used to calculate the specific fish growth rate (G) using the equation G = (ln L_t_−ln L_0_) / t_n_ [43], where ln L_t_ is the natural logarithm of fish length at 8 weeks, ln L_0_ is that of fish length at Time 0 and t_n_ is the duration of the experiment (8 weeks). We ranked fish in each tank by body length at Time 0 and t_n_ to identify fish individuals and be able to calculate G because our tagging equipment (e.g. elastomer) is not suitable for such small fish. Moreover, we calculated the Scaled Mass Index (SMI) as an index of body condition: SMI = W_i_ (L_o_/L_i_)^bSMA^ [44], where W_i_ and L_i_ are the weight and length of each fish individual, respectively, L_0_ is the arithmetic mean length of all the tested mosquitofish and b_SMA_ is the slope of a standardised major axis regression of the mass-length relationship. The SMI is regarded as a correlate of energy and fitness measures [44].

#### Energetic reserves

Changes in fish δ^13^C and δ^15^N stable isotope signatures and calorimetry were used as two complementary measures of energetic reserves. Stable isotopes are widely used in studies of trophic ecology because the isotopic composition of predator tissue is naturally altered by the type and amount of food assimilated [45]. Moreover, tissue isotopic differences are due to changes in metabolism, including increased lipid storage [46,47], because lipids are about 6–7% depleted in ^13^C relative to protein [48]. We used the δ^13^C and δ^15^N ratio as proxy for lipid content in white muscle because this is the tissue widely used in fisheries (e.g. [47,49]). However, lipid content varies amongst fish tissues [50], so that we used the caloric content of the whole fish as an additional measure of energy content.

For stable isotope analyses, we freeze-dried fish muscle samples from below the dorsal fin of 3 adult fish from each tank and we ground them to fine power. Two sub-samples of 0.30 mg each were placed into tin buckets and crimped for combustion to determine δ^13^C and δ^15^N using a Flash EA1112 and TC/EA coupled to a stable isotope mass spectrometer Delta C through a Conflo III interface (ThermoFinnigan). Analytical accuracy was controlled using replicate assays of certified standards indicating an analytical error of ±0.1‰ and ±0.3‰ for δ^13^C and δ^15^N, respectively. Isotope ratios are expressed conventionally as δ values in ppt (‰) according to the following equation: *δX* = ((*R*_sample_/*R*_standard_)– 1) ·1000, where *X* (‰) is ^13^C, ^15^N, and *R* are the corresponding ratios ^13^C/^12^C ^15^N/^14^N, related to the standard values: *R* standard for ^13^C is Pee Dee Belemnite, for ^15^N is atmospheric nitrogen.

For calorimetry, we used three adults per tank and we pooled all juveniles from each tank to reach the detection limits of the IKA Calorimeter c7000 (Germany). Samples were oven-dried at 60ºC for 48 h, weighted and the caloric content was expressed as joules per gram (J/g).

#### Histopathology

A random sample of three fish from each tank was processed for histology. The head and viscera of adult fish were fixed individually in 10% buffered formalin, dehydrated in ethanol, cleared in xylene and embedded in paraffin wax [51]. Juveniles were processed as a whole due to small size.

Sagittal sections of 5 μm thick were cut in all fish at the same position and stained with conventional haematoxylin-eosin [51]. Observations were made under an Olympus BH light microscope at 400x magnification. We focused on liver and gills because nitrate uptake is through gills [52] and liver is the target of many toxicants [27]. Gill alterations and the number of mucous cells in the gills were recorded and expressed as a ratio out of the 50 secondary lamellae and spaces examined. Liver alterations and the number of melanomacrophague centres were expressed as a ratio out of the number of microscope fields examined. We took photographs with a Jenoptik ProgRes C3 camera and used the ImageJ software to quantify the area (μm^2^) of perivisceral fat in 3 sections of each juvenile fish as an additional measure of energy storage. Outcomes were expressed as average per juvenile.

#### Feeding behaviour

**Fig 1:**
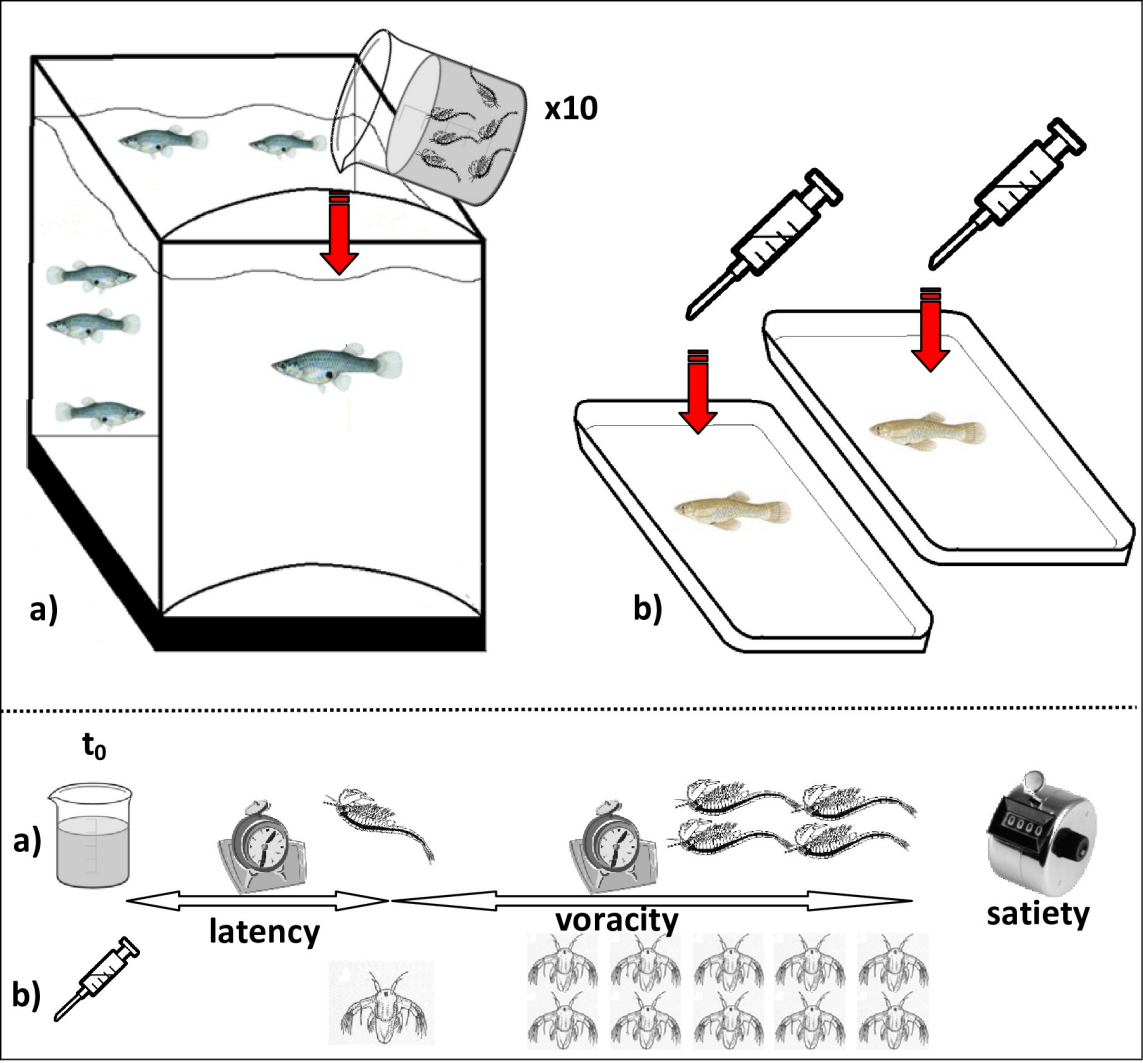
Experimental setting for the feeding behaviour assay of adults and juveniles of mosquitofish. a) 10 prey items are offered to an isolated adult mosquitofish. b) Live brine shrimp nauplii are offered with a syringe to an isolated juvenile mosquitofish in a tray. After adding food (t_0_) we quantified: feeding latency time (time to capture the first prey, t_LAT_), voracity time (time to capture 4 preys in adults and 10 in juveniles, t_VOR_) and satiety.

Temporal changes in feeding behaviour were quantified in all experimental fish, which were not fed 24 h before the assay to ensure all fish were hungry. The assay was conducted in an aquarium with a plastic sheet in one side, where the tested fish were left for 3 minutes to acclimatise before the trial (Fig 1). The trial began when the sheet was gently removed and ten size-matched unfrozen *Artemia* adults in 200 ml of water were gently released into the experimental area on the opposed side of the tested fish. Live *Artemia* nauplii were used for juveniles. The assay was repeated individually for all fish and the same observer, sat in front of the aquarium, recorded: i) latency defined as the time spent to capture the first item, ii) voracity defined as the time needed to capture the first items (four in adults and ten in juveniles), and iii) satiety defined as the total number of items eaten without stopping > 2 minutes. If a fish ate all 10 prey items, then we added *Artemia* individuals on the water surface until satiety.

### Statistical analyses

All analyses were conducted using the R software [53] and the functions outlined below. Spearman rank correlation coefficients were used to examine associations amongst all biomarker responses. The effects of chronic nitrate exposure on mortality, fish growth, SMI, caloric content, the C/N ratio, histopathological and behavioural measures were assessed using generalized linear mixed models (GLMMs). Data on males, females and juveniles were analysed separately because there were significant differences in all response variables (S5 Table). To avoid pseudoreplication, aquarium ID was included as random intercept in all models to account for the fact that fish were exposed to nitrate in batches (6 adults or 11 juveniles). For juveniles, aquarium ID was nested within mother ID as random intercept to account for systematic differences amongst clutches. Nitrate was included as fixed effect in all models. The interaction between nitrate and time was included in the behaviour model to test whether the effects of nitrate on fish varied with exposure time. The distribution of all response variables was visually inspected and the error distribution in GLMMs was chosen accordingly (e.g. Gaussian for fish growth, Poisson for the number of prey eaten). Model assumptions were checked by inspecting diagnostic plots of residuals [54]. The function *Anova* within the package *car* [55] was used to assess significance at P ≤ 0.05.

## Results

Mosquitofish were evenly distributed by size and sex amongst treatments and fish did not differ in size amongst aquaria at Time 0, either for males (F = 0.54; P = 0.90) or females (F = 1.22; P = 0.28). However, females (Mean ± S.E. = 37.6 ± 0.42 mm) were significantly bigger than males (25.9 ± 0.18 mm; t = 25.8; P < 0.001). There was no mortality in males due to nitrate and only minor mortality was recorded for juveniles (3.6%) and females (3.3%). Nonetheless, mortality did not differ significantly between treatments (Nitrate: χ^2^ = 1.59, P = 0.66) or sexes (Sex: χ^2^ = 0.43, P = 0.93).

### Growth, body condition and energetic reserves

An eight-week nitrate exposure did not alter significantly the growth rate or body condition, as defined by the SMI, of males, females and juveniles (Table 3). The caloric content of juveniles at 50 mg NO_3_^-^/l and in the controls at <10 mg NO_3_^-^/l was markedly higher than at 250 mg NO_3_^-^/l (Table 3). However, males significantly increased in caloric content at 50 mg NO_3_^-^/l compared to the other two concentrations. Females showed no significant differences amongst treatments (Table 3). Nitrate also did not affect the δ^13^C and δ^15^N measures in the white muscle of all tested fish (Table 3).

**Table 3:** Biomarkers used to appraise the health status (energy content, mass–length measures, histopathology) of males, females and juveniles of mosquitofish (*Gambusia holbrooki*) exposed to three experimental nitrate concentrations (<10 mg/l, 50 mg/l, 250 mg/l).

		Control		50 mg NO_3_^-^/l	250 mg NO_3_^-^/l
**Histopathology**	Juveniles *	MMC	0	0	0
		Lam%	6.8±1.2	5.7±1.7	5.8±1.2
		GMC	0.3±0.2	0.7±0.3	0.9±0.3
		PVFC	3.51E^5^±5.47E^4^	2.84E^5^±4.50E^4^	**2.18E**^**5**^**±1.62E**^**4**^
	Males	MMC	1.1±0.5	0.9±0.2	1.2±0.2
		Lam%	1.6±0.6	1.6±0.6	0.7±0.3
		GMC	0.3±0.2	0.5±0.2	0.7±0.3
	Females	MMC	15.5±7.3	8.0±1.8	23.7±12.3
		Lam%	7.5±2.2	8.8±3.3	9.7 ±2.1
		GMC	2.1±0.6	2.3±0.8	2.2±0.6
**Calorimetry**	Juveniles	J/g	23008.5±279.8	22540.2±547.0	**21895.1±483.1**
	Males	J/g	15820.8±832.3	**17920.3±403.3**	16165.0±625.9
	Females	J/g	18471.4±409.2	18770.9±218.5	18696.0±171.4
Stable isotope analysis **	Males	δ^13^C	-24.1±0.2	-24.5±0.2	-24.5±0.1
		δ^15^N	13.3±0.4	13.4±0.4	13.0±0.2
		C/N_m_	4.29±0.06	4.32±0.08	4.27±0.05
	Females	δ^13^C	-21.3±0.1	-21.2±0.1	-21.3±0.1
		δ^15^N	15.8±0.3	15.5±0.2	15.6±0.2
		C/N_m_	3.97±0.03	3.96±0.02	4.00±0.03
**Mass–length measures**	Juveniles	SMI	0.192±0.002	0.189±0.002	0.189±0.002
		G	1.11E^-2^±3.17E^-4^	1.16E^-2^±2.71E^-4^	1.06E^-2^±2.40E^-4^
	Males	SMI	0.188±0.006	0.193±0.004	0.217±0.012
		G	6.21E^-4^±1.23E^-4^	6.90E^-4^±1.16E^-4^	6.39E^-4^±1.40E^-4^
	Females	SMI	0.193±0.002	0.192±0.003	0.193±0.002
		G	6.48E^-4^±9.32E^-5^	7.15E^-4^±6.79E^-5^	6.86E^-4^±7.47E^-5^

Values represent mean ± standard Error. MMC: melanomacrophague centers per microscope field; Lam%: % of gill secondary lamellae with alterations; GMC: number of gill mucous cells per section. PVFC: perivisceral fat content as μm^2^ per section; J/g: energy density; δ^13^C/δ^15^N: carbon or nitrogen fractionation; C/N_m_: molar carbon to nitrogen ratio; SMI: scaled mass index, computed using fresh weight including viscera; G: specific growth rate. Bold values indicate a significant effect compared to control values at α = 0.05.

* Perivisceral fat content was analysed only in juveniles because the quantification method we used was only reliable in sagittal sections of the whole body.

** Stable isotopes in juveniles were not analysed.

### Histopathology

A detailed examination of all slides did not reveal overt clinical signs of disease, but some tissular changes were observed in liver and gills (Table 3, S2 and S3 Figs). Occasional telangiectasia and slight epithelial lifting were observed in secondary lamellae. However, we did not observe other tissue alterations such as hyperplasia, hypertrophy or increased number of mucous cells. All liver samples had regularly aligned cords of hepatocytes (S3 Fig). However, slight changes in the staining intensity of the cytoplasm were observed in liver tissue, probably related to glycogen deposits and occasional lipid droplets. The presence of macrophage aggregates was restricted to adult fish, with females having a larger number than males, but without significant changes due to nitrate (Table 3). The amount of perivisceral adipose tissue in juveniles at the highest nitrate concentration was lower than in juveniles in the other treatments (Table 3, S4 Table).

### Feeding behaviour

**Fig 2:**
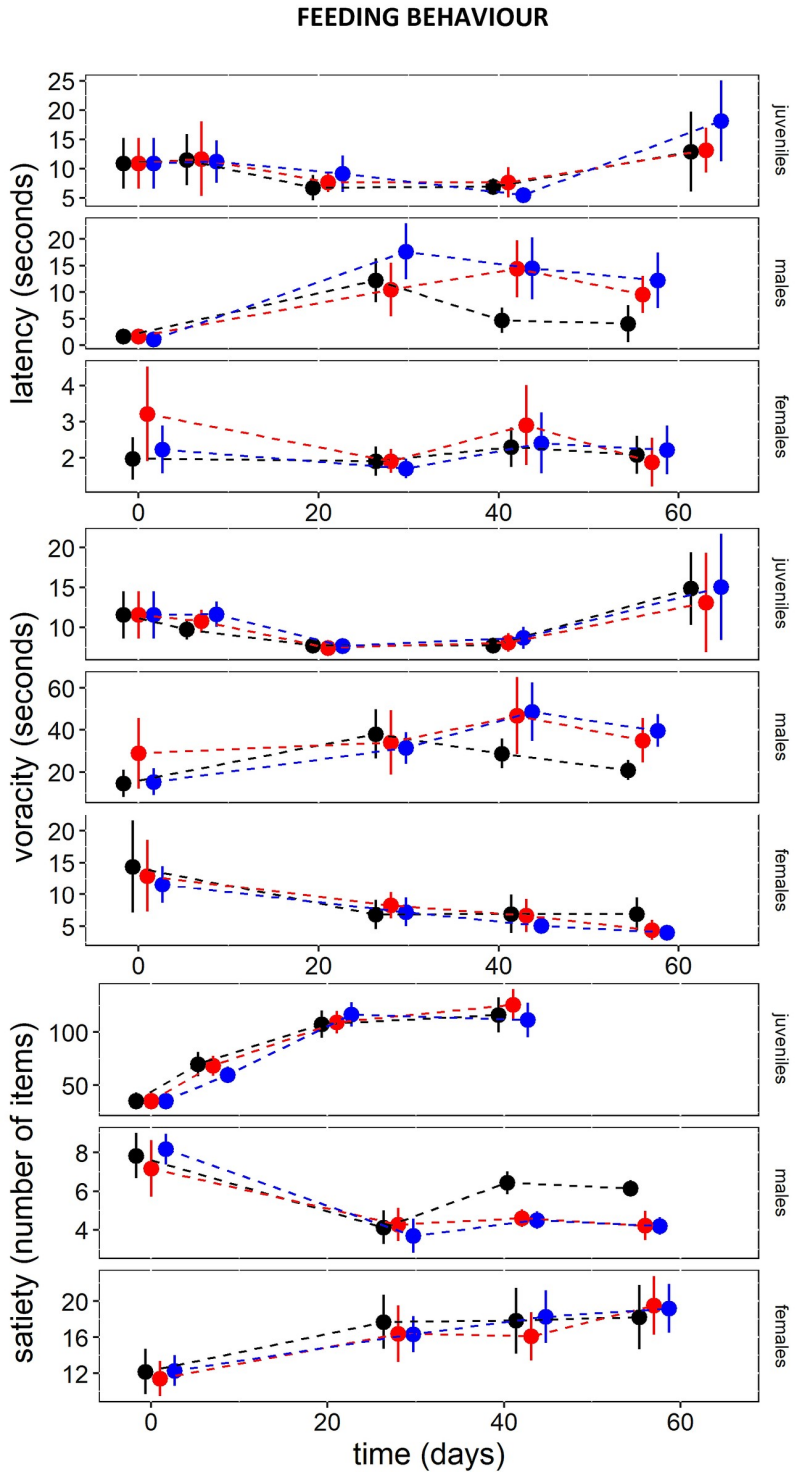
Feeding behaviour variables along the experiment. Symbols and bars represent means ± 95% confidence intervals for each variable and assigned for each treatment (black: control, red: 50 mg NO_3_^-^/l, blue: 250 mg NO_3_^-^/l). a) Latency is the time spent to capture the first food item. b) Voracity is the time to capture a given number of food items. c) Satiety is the total number of eaten food items.

Males, females and juveniles showed differences in latency, voracity and satiety, but without significant changes due to nitrate apart from males from the sixth week onwards (Fig 2, S1 Table). Males at 50 and 250 mg NO_3_^-^/l exhibited lower satiety values and lower voracity (i.e. > time to capture prey) than those in the controls at <10 mg NO_3_^-^/l (Fig 2). Overall, females and juveniles had a higher voracity and satiety than males, and juveniles had greater latency times than adults (Fig 2). Females and juveniles also tended to increase voracity and satiety and to reduce latency times throughout the experiment compared to males (Fig 2).

### Pair-wise correlations amongst biomarkers

Spearman rank correlation coefficients were generally low amongst all biomarkers measured in juveniles, males and females of mosquitofish. There was a strong positive correlation between calorimetry and growth (ρ = 0.73, P = 0.002) and between calorimetry and the amount of perivisceral fat cells in juveniles (ρ = 0.72, P = 0.003, S6 Table). However, a marked negative correlation was found between satiety and latency time of males (ρ = -0.78, P <0.001, S7 Table). A strong negative association was observed in females for d^15^N and growth (ρ = -0.74, P = 0.002), even though the relationship was positive between C/N and growth (ρ = 0.77, P <0.001, S8 Table).

## Discussion

This is the first comprehensive study examining the chronic effects of nitrate on a widely introduced fish species, as exemplified by the eastern mosquitofish (*Gambusia holbrooki*) [28]. Moreover, this is one of the few ecotoxicological studies using short- and long-term biomarkers (e.g. growth, histopathology, feeding assays) in females, males and juveniles of the same species. Overall, we did not find overt clinical signs of disease, which supports the prevailing idea that many invasive species, including mosquitofish, have wide tolerance to changes in water quality [28,56]. However, the fact that nitrate altered food intake or energetic reserves in males and juveniles suggests that concentrations >50 mg NO_3_^-^/l cannot be considered completely safe [11,21].

Many studies have shown that males are more likely to acquire diseases than females, including fish [37,57–59]. We did not find gross pathological alterations in any fish, but the more marked effects of nitrate on males provide some support for males being the sicker sex [35,36]. The weaker response of the tested fish to nitrate is unlikely to be much attributed to pre-acclimation to nitrate at the collection site (9.9 ± 3.0 mg NO_3_^-^/l), as reported for amphibians [33]. However, this outcome does not exclude the possibility of nitrate tolerance being increased due to pre-exposure to other ions, including those of water hardness (see [60]), which is high at the collection site and in the laboratory dechlorinated tap water due to a calcareous geology. The effects of metals and chlorine compounds on fish in the animal facility probably were negligible because tap water is filtered through active charcoal and the aquarium product Sera Aquatan is used to further guarantee water is free of metals and chlorine (see [39]). The fact that juveniles had a greater tolerance to nitrate than males was unexpected because young fish are generally more sensitive to chronic pollution than adults [26,38]. However, 96-h LC50 tests revealed that susceptibility to nitrate increases with body size in the Siberian sturgeon *Acipenser baeri* [61]. Although we cannot reveal the mechanisms for the mild effects of nitrate on juveniles because nitrate metabolites in tissues were not measured (e.g. nitric oxide [62]), differences in space and behaviour between adults and juveniles might partially explain outcomes. Adults were kept at lower densities (6 fish per tank) than juveniles (11 fish per tank) and not surprisingly, we observed more agonistic interactions among adults due to confinement.

Mosquitofish populations are often confined in small water bodies and female-biased [63,64], including in the collection site of the studied fish (authors *pers*. *observ*.). The biased sex-ratio has been attributed to the high life-span of females compared to males [65]. We built on this knowledge by showing that females may dominate in number because they are more tolerant than males to polluted waters, where mosquitofish often occur (e.g. [66]). Female mosquitofish had higher feeding rates than males regardless of the nitrate treatment, which is consistent with previous data in clean water [64]. Given that higher nitrogen excretion rates have been reported in females [64], it is possible that tolerance to environmental nitrate can be predicted from nitrogen excretion rates in fish. Nonetheless, sensitivity to nitrate probably depends on many factors in wild fish, including temperature, predation, and the fact that a parasite with more severe effects on males than females [37] is more sensitive to nitrate than the fish host [32]. In contrast to mainstream literature in which external factors other than pollutants are often not included in toxicological assays [27], our study accounted for intraspecific interactions; that is, several individuals were exposed to nitrate in the same tank instead of fish being exposed individually. Agonistic interactions probably are amongst the most important factors to explain *G*. *holbrooki* performance alongside sex because females are more aggressive towards conspecifics than males [67].This might explain why more females died during the experiment than males, although mortality did not differ significantly amongst treatments.

Feeding traits were more affected by nitrate than other biomarkers measured in male mosquitofish, which supports that food intake is amongst the most sensitive biomarkers in ecotoxicology [68,69]. Although we cannot identify the mechanisms, it might be a response-mediated by stress hormones (e.g. cortisol) because high levels of these hormones often reduce appetite in fish and other animals [70]. However, cortisol levels remained stable in females of Siberian sturgeon (*Acipenser baeri*) after 30-day exposure to 250 mg NO_3_^-^/l, as opposed to the reproductive hormones testosterone and estradiol [24]. Moreover, there is correlative evidence for reduced sperm count in male mosquitofish at < 22 mg NO_3_^-^/l [29]. These studies illustrate that nitrate is an endocrine disruptor through *in-vivo* conversion to nitric oxide, which is involved in many metabolic pathways [71], suggesting that it is possible that the effects of nitrate on fish probably would have been stronger than observed if we had used biochemical biomarkers. Nevertheless, many biochemical alterations often do not have far reaching impacts on individuals, reason for which they are considered of less ecological relevance than behavioural assays, including the feeding traits we measured [27].

Even though fish differed in food intake amongst nitrate treatments, we did not observe overt signs of disease, including reduced fish growth. Reduced food ingestion in males may be attributed to fatigue because nitrate forms methaemoglobin, which transports oxygen worse than haemoglobin [19]. However, fish can cope with moderate methaemoglobinemia [72], especially in hard water, such as ours in the laboratory, which may have mitigated nitrate adverse effects [60]. Growth was expected to decrease in mosquitofish because iodine uptake, which is needed for thyroid functions and animal development, is altered by nitrate, but concentrations up to 11 mg NO_3_^-^/l did not impair the thyroid function in perch (*Perca fluviatilis*) and Crucian carp (*Carassius carassius*) [73]. The neutral effect of nitrate we saw on mosquitofish growth agrees with Freitag et al. [74], who found that concentrations up to 450 mg NO_3_^-^/l had no effect on the thyroid hormone levels in Atlantic salmon (*Salmo salar*). Our outcome is also consistent with studies in other freshwater taxa showing that nitrate effects on growth and survival occur at > 500 mg NO_3_^-^/l (e.g. [31,75–77]). However, the neutral effect of nitrate on mosquitofish does not exclude the possibility that fish exposed to nitrate may reduce their ability to cope with other pollutants if nitrate alters the internal ionic composition of fish at an osmoregulation cost and probably impairs important enzymatic complexes, such as those involved in detoxification [62].

Histopathological analyses revealed no relevant tissue alterations because slight epithelial lifting and other alterations we saw are not pathological but tissue processing artifacts (see [78]). Changes in caloric content only matched with histological data for juveniles at 250 mg NO_3_^-^/l, which reduced energy reserves as also exemplified by peripheral fat content, but no major changes in δ^13^C and δ^15^N measures of muscle occurred in fish from any treatment combination. These findings suggest that no single tissue can be a good proxy of overall fish energy reserves because they vary greatly amongst tissues [50]. Moreover, our findings confirmed that no biomarker, including mass-length relationship indices such as the SMI, can be assumed to accurately reflect ‘true condition’ without analysing body composition [27,44]. The lack of response of the SMI may be attributed to the fact that nitrate did not markedly change fish weight, possibly because although fish reduced food intake, fish were fed daily until satiety. However, the biochemical composition of fish tissues might have changed due to nitrate because pollutants often alter tissue stoichiometry [52] with potential far reaching impacts for fish predators. In this regard, juvenile mosquitofish altered energy content in tissues, but food intake or growth were not affected, which suggests that growth is prioritised over lipid storage, probably to reduce size-dependent predation mortality [79].

In our study, energy costs are likely to be mostly attributed to intraspecific interactions and osmoregulation due to nitrate. Osmoregulation cost probably was caused mainly by the anion nitrate (NO_3_^-^, see [80]) and, to a minor degree, by the cation sodium (Na^+^) of the salt (NaNO_3_). The sodium concentration in the highest nitrate concentration was below 1 parts per thousand (ppt) and there is no experimental evidence for major changes in mosquitofish metabolism at 20 ppt [81] or in mosquitofish plasma osmotic concentration at 10 ppt [82]. Surprisingly, we found that the caloric content of males was higher at 50 mg NO_3_^-^/l than at 250 mg NO_3_^-^/l and in the controls at <10 mg NO_3_^-^/l. Reduced caloric content in males at 250 mg NO_3_^-^/l compared to 50 mg NO_3_^-^/l can be due to osmoregulation cost increasing nitrate concentration. However, this rationale does not explain why males at 250 mg NO_3_^-^/l had a similar caloric content to those in the controls at <10 mg NO_3_^-^/l, although the latter had the highest feeding rates in the study. Less energy stored implies that males at <10 mg NO_3_^-^/l had an additional cost than those at 50 mg NO_3_^-^/l, which may be the courtship display. Control males were reproductive active and we observed copulation attempts with other males, a behaviour that often occurs in male poeciliids in the absence of females [83]. Courtship display has an energetic cost [84,85], which probably was reduced at 50 mg NO_3_^-^/l because nitrate, even at lower concentrations, reduces testosterone [62,86] and this hormone promotes the sexual characteristics of males.

### Conclusions

Our study shows that females of the invasive fish *G*. *holbrooki* are more tolerant to nitrate pollution than males and juveniles, but that there are all weak effects combining short- and long-term biomarkers. Therefore, the ecological impact of this invasive fish seems not to be much affected by nitrate pollution, especially if populations are female-biased. However, our study cannot inform the indirect effects that nitrate may have on *G*. *holbrooki* through the alteration of aquatic food-webs, including a possible reduction in prey numbers accompanied by impaired food intake in males. There is the pressing need for an–omics screening (e.g. transcriptomics) to identify simultaneously all the metabolic pathways that are altered in fish exposed to nitrate in order to improve the mechanistic understanding of the effects of this widely distributed subsidy and pollutant in aquatic ecosystems.

## Supporting information

S1 Table Mixed models analysis of variance of satiety, latency to eat and voracity of juveniles, males and females along the experiment. (PDF)

S2 Table Mixed models analysis of variance of calorimetry and stable isotopes of juveniles, males and females at the end of the experiment. (PDF)

S3 Table Mixed models analysis of variance of growth and body condition of juveniles, males and females at the end of the experiment. (PDF)

S4 Table Mixed models analysis of variance of histopathology variables of juveniles, males and females at the end of the experiment. (PDF)

S5 Table Mixed models analysis of variance of all biomarkers with sex and length as principal explicative variables. (PDF)

S6 Table Spearman rank correlation coefficients examining the associations amongst all biomarkers in juveniles. (PDF)

S7 Table Spearman rank correlation coefficients examining the associations amongst all biomarkers in males. (PDF)

S8 Table Spearman rank correlation coefficients examining the associations amongst all biomarkers in females. (PDF)

S9 Table Mean (±Standard Error) of the variables used to appraise the feeding rates of males, females and juveniles of mosquitofish. (PDF)

S1 Fig Histological samples of the abdominal cavity. (PDF)

S2 Fig Histological samples of the gill tissue examined. (PDF)

S3 Fig Histological samples of the hepatic tissue. (PDF)

S1 File Compressed file including separate datasets for juveniles, females and males data. (7Z)
