# Supplementary material for: Ecologically relevant biomarkers reveal that chronic effects of nitrate depend on sex and life stage in the invasive fish Gambusia holbrooki
Source: PLoS One. 2019 Jan 28;14(1):e0211389. doi: 10.1371/journal.pone.0211389 (PMC6349331; doi:10.1371/journal.pone.0211389)
Supplement: S1 Table — (PDF) [file pone.0211389.s001.pdf]

S1 Table. Mixed models analysis of variance of satiety, latency to eat and voracity of juveniles, males and females along the experiment. Asterisk (*) denotes significant factors and interactions at α = 0.05. Satiety was square-root transformed except for males, while latency and voracity was log_e transformed in all fish.

Function	Sex		Effect size		t-value	P
Satiety	J	Intercept	11.1±0.40	*	27.7	<0.001
		time	-0.012±0.019		-0.651	0.515
		$50NO_3^-$	-0.089±0.328		-0.271	0.793
		$250NO_3^-$	-0.524±0.332		-1.577	0.149
		$time^2$	$-4.8E^{-3}\pm8.2E^{-4}$	*	-5.888	<0.001
		$time{:}50NO_3^-$	0.012±0.018		0.678	0.498
		$time{:}250NO_3^-$	$7.1E^{-3}\pm0.018$		0.386	0.700
	M	Intercept	5.21±0.32	*	16.1	<0.001
		time	0.05±0.01	*	3.42	<0.001
		$50NO_3^-$	-1.12±0.44	*	-2.52	0.027
		$250NO_3^-$	-1.31±0.44	*	-2.94	0.012
		$time^2$	$2.2E^{-3}\pm3.8E^{-4}$	*	5.81	<0.001
		$time{:}50NO_3^-$	-0.059±0.019	*	-3.19	0.002
		$time{:}250NO_3^-$	-0.057±0.019	*	-3.08	0.002
	F	Intercept	3.38±0.30	*	11.1	<0.001
		time	0.015±0.004	*	3.90	<0.001
		$50NO_3^-$	-0.063±0.432		-0.146	0.886
		$250NO_3^-$	0.088±0.431		0.205	0.841
		$time{:}50NO_3^-$	$1.2E^{-3}\pm5.6E^{-3}$		0.211	0.833
		$time{:}250NO_3$	$9.5E^{-4}\pm5.5E^{-3}$		0.172	0.864
Feeding latency	J	Intercept	1.62±0.12	*	13.4	<0.001
		time	$-5.5E^{-3}\pm2.9E^{-3}$		-1.906	0.057
		$50NO_3^-$	0.053±0.116		0.463	0.643
		$250NO_3^-$	0.112±0.120		0.934	0.351
		$time^2$	$5.1E^{-4}\pm9.7E^{-5}$	*	5.18	<0.001
		$time{:}50NO_3^-$	$6.6E^{-3}\pm3.8E^{-3}$		1.75	0.080
		$time{:}250NO_3$	$4.1E^{-3}\pm3.7E^{-3}$		1.097	0.273
	M	Intercept	1.72±0.13	*	13.04	<0.001
		time	-0.026±0.007	*	-3.68	<0.001
		$50NO_3^-$	0.52±0.18	*	2.92	0.013
		$250NO_3^-$	0.65±0.18	*	3.68	0.003
		$time^2$	$-1.1E^{-3}\pm1.9E^{-4}$	*	-5.54	<0.001
		$time{:}50NO_3^-$	0.027±0.010	*	2.86	0.005
		$time{:}250NO_3$	0.020±0.010	*	2.09	0.038
	F	Intercept	0.47±0.12	*	3.91	<0.001
		time	$2.1E^{-3}\pm2.7E^{-3}$		0.764	0.446
		$50NO_3^-$	0.32±0.17		1.90	0.082
		$250NO_3^-$	0.10±0.17		0.591	0.565
		$time{:}50NO_3^-$	$-7.3E^{-3}\pm3.8E^{-3}$		-1.91	0.058
		$time{:}250NO_3$	$-2.8E^{-3}\pm3.8E^{-3}$		-0.74	0.458

Voracity	J**	Intercept	1.990±0.042	*	46.9	<0.001
		time	$-1.8E^{-3}±1.4E^{-3}$		-1.30	0.193
		$50NO_3^-$	-0.032±0.047		-0.676	0.500
		$250NO_3^-$	0.047±0.048		0.986	0.325
		$time^2$	$3.4E^{-4}±4.6E^{-5}$	*	7.38	<0.001
		$time:50NO_3^-$	$-3.8E^{-3}±1.9E^{-3}$		-2.02	0.044
		$time:250NO_3$	$-2.1E^{-3}±1.9E^{-3}$		-1.12	0.264
	M	Intercept	3.17±0.13	*	24.13	<0.001
		time	$-9.4E^{-3}±5.6E^{-3}$		-1.66	0.098
		$50NO_3^-$	0.206±0.180		1.14	0.276
		$250NO_3^-$	0.339±0.179		1.89	0.083
		$time^2$	$-3.1E^{-4}±1.6E^{-4}$	*	-1.97	<0.050
		$time:50NO_3^-$	$9.5E^{-3}±7.6E^{-3}$		1.25	0.214
		$time:250NO_3$	0.021±0.008	*	2.83	0.005
	F	Intercept	2.17±0.16	*	13.7	<0.001
		time	-0.011±0.003	*	-3.65	<0.001
		$50NO_3^-$	0.14±0.22		0.640	0.534
		$250NO_3^-$	0.048±0.219		0.221	0.829
		$time:50NO_3^-$	$-6.3E^{-3}±4.4E^{-3}$		-1.44	0.152
		$time:250NO_3$	$-5.5E^{-3}±4.3E^{-3}$		-1.28	0.201

** Although the t-test gives significant results for the interaction $time:50NO_3^-$ in juveniles, the overall F-test gave P=0.129.
