# Supplementary material for: Ecologically relevant biomarkers reveal that chronic effects of nitrate depend on sex and life stage in the invasive fish Gambusia holbrooki
Source: PLoS One. 2019 Jan 28;14(1):e0211389. doi: 10.1371/journal.pone.0211389 (PMC6349331; doi:10.1371/journal.pone.0211389)
Supplement: S2 Table — (PDF) [file pone.0211389.s002.pdf]

S2 Table. Mixed models analysis of variance of calorimetry and stable isotopes of juveniles, males and females at the end of the experiment. The following abbreviatures are used: J/g, energy density; $\delta^{13}C/\delta^{15}N$, carbon or nitrogen fractionation; C/N_m, molar carbon to nitrogen ratio. Asterisk (*) denotes significant factors and interactions at $\alpha = 0.05$.

Function	Sex	Effect size			t-value	P
J/g	J	Intercept	23132±463	*	49.95	<0.001
		$50NO_3^-$	-706±381		-1.85	0.101
		$250NO_3^-$	-1366±391	*	-3.50	0.008
	M	Intercept	15084±838	*	18.0	<0.001
		$50NO_3^-$	2114±937	*	2.26	0.044
		$250NO_3^-$	335±1054		0.32	0.756
	F	Intercept	18471±409	*	45.14	<0.001
		$50NO_3^-$	299±464		0.645	0.531
		$250NO_3^-$	225±444		0.506	0.622
$\delta^{13}C$	M	Intercept	-24.11±0.23	*	-106.4	<0.001
		$50NO_3^-$	-0.43±0.32		-1.35	0.201
		$250NO_3^-$	-0.37±0.32		1.17	0.267
	F	Intercept	-21.27±0.06	*	-371.3	<0.001
		$50NO_3^-$	0.08±0.10		0.79	0.447
		$250NO_3^-$	-0.07±0.11		-0.66	0.524
$\delta^{15}N$	M	Intercept	13.33±0.39	*	34.0	<0.001
		$50NO_3^-$	0.09±0.55		0.16	0.878
		$250NO_3^-$	-0.37±0.55		-0.66	0.521
	F	Intercept	15.78±0.28	*	55.9	<0.001
		$50NO_3^-$	-0.29±0.40		-0.74	0.476
		$250NO_3^-$	-0.20±0.40		-0.50	0.625
C/N_m	M	Intercept	4.29±0.07	*	65.6	<0.001
		$50NO_3^-$	0.03±0.09		0.34	0.740
		$250NO_3^-$	-0.01±0.09		-0.13	0.901
	F	Intercept	3.97±0.03	*	115.4	<0.001
		$50NO_3^-$	-0.02±0.05		-0.35	0.731
		$250NO_3^-$	0.02±0.05		0.49	0.630
