# Supplementary material for: Ecologically relevant biomarkers reveal that chronic effects of nitrate depend on sex and life stage in the invasive fish Gambusia holbrooki
Source: PLoS One. 2019 Jan 28;14(1):e0211389. doi: 10.1371/journal.pone.0211389 (PMC6349331; doi:10.1371/journal.pone.0211389)
Supplement: S3 Table — (PDF) [file pone.0211389.s003.pdf]

S3 Table. Mixed models analysis of variance of growth and body condition of juveniles, males and females at the end of the experiment. The following abbreviatures are used: SMI_t: scaled mass index, computed using fresh weight including viscera; G: specific growth rate. Asterisk (*) denotes significant factors and interactions at α = 0.05.

Function	Sex	Effect size			t-value	P
G	J	Intercept	$1.1E^{-2}±3.8E^{-4}$	*	29.61	<0.001
		$50NO_3^-$	$2.0E^{-4}±4.1E^{-4}$		0.49	0.640
		$250NO_3^-$	$-6.6E^{-4}±4.1E^{-4}$		-1.61	0.145
	M	Intercept	$6.2E^{-4}±1.5E^{-4}$	*	4.19	<0.001
		$50NO_3^-$	$6.9E^{-5}±2.1E^{-4}$		0.33	0.748
		$250NO_3^-$	$1.9E^{-5}±2.1E^{-4}$		0.09	0.931
	F	Intercept	$6.5E^{-4}±1.2E^{-4}$	*	5.19	<0.001
		$50NO_3^-$	$6.1E^{-5}±1.8E^{-4}$		0.34	0.736
		$250NO_3^-$	$3.2E^{-5}±1.8E^{-4}$		0.18	0.862
**SMI_t**	J	Intercept	0.193±0.004	*	49.60	<0.001
		$50NO_3^-$	-0.004±0.003		-1.25	0.248
		$250NO_3^-$	-0.006±0.003		-2.05	0.074
	M	Intercept	0.188±0.006	*	31.6	<0.001
		$50NO_3^-$	0.004±0.007		0.62	0.548
		$250NO_3^-$	0.028±0.013	*	2.18	<0.050
	F	Intercept	0.193±0.003	*	74.57	<0.001
		$50NO_3^-$	-0.001±0.004		-0.159	0.876
		$250NO_3^-$	0.000±0.004		0.068	0.947
