# Supplementary material for: Ecologically relevant biomarkers reveal that chronic effects of nitrate depend on sex and life stage in the invasive fish Gambusia holbrooki
Source: PLoS One. 2019 Jan 28;14(1):e0211389. doi: 10.1371/journal.pone.0211389 (PMC6349331; doi:10.1371/journal.pone.0211389)
Supplement: S4 Table — (PDF) [file pone.0211389.s004.pdf]

S4 Table. Mixed models analysis of variance of histopathology variables of juveniles, males and females at the end of the experiment. The following abbreviatures are used: MMC, melanomacrophagues centers; MF, microscope fields. Asterisk (*) denotes significant factors and interactions at α = 0.05.

Function	Sex		Effect size		t-value	P
MMC **	M	Intercept	0.809±0.509		1.59	0.126
		$50NO_3^-$	0.045±0.400		0.11	0.913
		$250NO_3^-$	0.334±0.360		0.93	0.372
		MF	0.114±0.083		1.37	0.185
	F	Intercept	3.835±0.500	*	7.68	<0.001
		$50NO_3^-$	-0.202±0.495		-0.41	0.691
		$250NO_3^-$	0.401±0.429		0.93	0.368
		MF	-0.022±0.073		-0.30	0.765
Gill secondary lamellae Alterations ***	J	Intercept	-0.328±0.503		-0.65	0.520
		$50NO_3^-$	-0.162±0.300		-0.54	0.603
		$250NO_3^-$	-0.042±0.297		-0.14	0.890
	M	Intercept	-1.258±1.584		-0.79	0.434
		$50NO_3^-$	-0.056±0.663		-0.08	0.934
		$250NO_3^-$	-0.703±0.713		-0.99	0.344
	F	Intercept	1.317±0.325	*	4.06	<0.001
		$50NO_3^-$	0.164±0.442		0.37	0.716
		$250NO_3^-$	0.265±0.432		0.61	0.551
Gill mucous cells	J	Intercept	-1.386±0.646	*	-2.15	0.032
		$50NO_3^-$	0.693±0.847		0.82	0.413
		$250NO_3^-$	1.520±0.827		1.84	0.066
	M	Intercept	-1.386±0.646	*	-2.15	0.032
		$50NO_3^-$	1.099±0.842		1.31	0.192
		$250NO_3^-$	0.693±0.847		0.82	0.413
	F	Intercept	1.012±0.584		1.73	0.083
		$50NO_3^-$	0.288±0.875		0.33	0.742
		$250NO_3^-$	-0.319±0.801		-0.40	0.691
Perivisceral fat content	J	Intercept	12.68±0.15	*	83.65	<0.001
		$50NO_3^-$	-0.216±0.143		-1.518	0.168
		$250NO_3^-$	-0.386±0.146	*	-2.643	0.030

** Melanomacrophagues centers were not present in juveniles and no statistical model was computed.

*** In gill mucous cells analysis it was necessary to do a zero-inflated model due to excess of zeros. The values shown are the statistics for the binomial part (models the absence and presence of 0’s). The counts are not shown for easiness of the reader; no statistical differences occurred between treatments.
