# Supplementary material for: Ecologically relevant biomarkers reveal that chronic effects of nitrate depend on sex and life stage in the invasive fish Gambusia holbrooki
Source: PLoS One. 2019 Jan 28;14(1):e0211389. doi: 10.1371/journal.pone.0211389 (PMC6349331; doi:10.1371/journal.pone.0211389)
Supplement: S5 Table — (PDF) [file pone.0211389.s005.pdf]

S5 Table. Mixed models analysis of variance of all biomarkers with sex and length as principal explicative variables. The following abbreviatures are used: MMC, melanomacrophagues centers; MF, microscope fields; 2Lam%: % of gill secondary lamellae with alterations; GMC: number of gill mucous cells; J/g, energy density; $\delta^{13}C/\delta^{15}N$, carbon or nitrogen fractionation; C/N_m, molar carbon to nitrogen ratio; SMI_t: scaled mass index, computed using fresh weight including viscera; G: specific growth rate.

Function	Param.	F-value			P
**Histopathology**	MMC	Sex	40.28	*	<0.001
		length	192.16	*	<0.001
		MF	53.17	*	<0.001
	2Lam%	Sex	101.01	*	<0.001
		length	1.96		0.161
		lamellae	15.03	*	<0.001
	GMC	Sex	11.39	*	0.003
		length	9.62	*	0.002
**Calorimetry**	J/g	Sex	59.85	*	<0.001
		length	0.00		0.965
**Stable isotopes**	$\delta^{13}C$	Sex	120.75	*	<0.001
		length	1.32		0.253
	$\delta^{15}N$	Sex	4.640	*	0.034
		length	3.483		0.065
	C/N_m	Sex	3.88		0.052
		length	1.06		0.306
**Mass – length variables**	SMI	Sex	40.18	*	<0.001
		length	84.53	*	<0.001
	G	Sex	309.35	*	<0.001
		length	5.30	*	0.022
**Feeding Behaviour ****	Satiety	Sex	1617.47	*	<0.001
		length	59.25	*	0.011
		time	87.67.3	*	<0.001
		time:Sex	54.84	*	<0.001
	Latency	Sex	3.69	*	0.025
		length	0.57		0.450
		time	1.85		0.174
		time:Sex	1.32		0.267
	Voracity	Sex	443.91	*	<0.001
		length	9.38	*	0.002
		time	8.36	*	0.004
		time:Sex	38.17	*	<0.001

** Satiety was square-root transformed; latency and voracity was log-transformed.
