# Supplementary material for: Ecologically relevant biomarkers reveal that chronic effects of nitrate depend on sex and life stage in the invasive fish Gambusia holbrooki
Source: PLoS One. 2019 Jan 28;14(1):e0211389. doi: 10.1371/journal.pone.0211389 (PMC6349331; doi:10.1371/journal.pone.0211389)
Supplement: S9 Table — (PDF) [file pone.0211389.s009.pdf]

S9 Table. Mean (±Standard Error) of the variables used to appraise the feeding rates of males, females and juveniles of mosquitofish. Juveniles had an extra measurement (T1) to account for feeding changes in rapidly growing fish. T0 in juveniles was measured with a different non-experimental set of fish to avoid extra manipulation.

Function	Sex	NO_3^-	T0	T1	T2	T3	T4
Latency time	J	Control		11.5±2.2	6.7±1.1	6.9±0.7	12.9±3.5
		50	10.9±2.2	11.7±3.3	7.6±0.8	7.7±1.3	13.2±2.0
		250		11.2±1.9	9.1±1.6	5.4±0.5	18.2±3.5
	M	Control	1.7±0.3		12.2±2.1	4.7±1.2	4.1±1.8
		50	1.7±0.6	No data	10.5±2.6	14.4±2.7	9.6±1.8
		250	1.2±0.4		17.7±2.7	14.5±3.0	12.2±2.7
	F	Control	2.0±0.3		1.9±0.2	2.3±0.3	2.1±0.3
		50	3.2±0.7	No data	1.9±0.2	2.9±0.6	1.9±0.3
		250	2.2±0.3		1.7±0.1	2.4±0.4	2.2±0.3
Voracity time	J	Control		9.8±0.7	7.7±0.5	7.7±0.3	14.9±2.3
		50	11.5±1.5	10.8±0.7	7.4±0.4	8.1±0.6	13.1±3.2
		250		11.6±0.8	7.6±0.3	8.7±0.7	15.1±3.4
	M	Control	14.5±3.3		38.1±6.0	28.8±3.6	20.9±2.4
		50	28.9±8.6	No data	34.0±7.8	46.9±9.3	35.1±5.4
		250	15.3±3.3		31.5±3.8	48.7±7.1	39.7±3.9
	F	Control	14.4±3.7		6.8±1.2	6.9±1.5	6.9±1.3
		50	12.9±2.9	No data	8.3±1.0	6.7±1.3	4.4±0.8
		250	11.6±1.5		7.2±1.1	5.1±0.6	4.0±0.4
Satiety	J	Control		69.7±5.8	107.5±6.6	116.3±8.4	
		50	35.4±4.0	68.3±4.8	109.4±5.4	125.8±7.5	No data
		250		60.0±4.0	116.5±5.9	111.5±8.3	
	M	Control	7.8±0.6		4.1±0.4	6.4±0.3	6.1±0.2
		50	7.2±0.7	No data	4.3±0.4	4.6±0.2	4.2±0.4
		250	8.2±0.4		3.7±0.5	4.5±0.2	4.2±0.2
	F	Control	12.2±1.3		17.7±1.5	17.8±1.9	18.2±1.8
		50	11.4±1.0	No data	16.4±1.6	16.1±1.4	19.5±1.7
		250	12.3±0.9		16.3±1.0	18.3±1.5	19.2±1.4
