# Supplementary material for: Ecologically relevant biomarkers reveal that chronic effects of nitrate depend on sex and life stage in the invasive fish Gambusia holbrooki
Source: PLoS One. 2019 Jan 28;14(1):e0211389. doi: 10.1371/journal.pone.0211389 (PMC6349331; doi:10.1371/journal.pone.0211389)
Supplement: S1 Fig — (PDF) [file pone.0211389.s010.pdf]

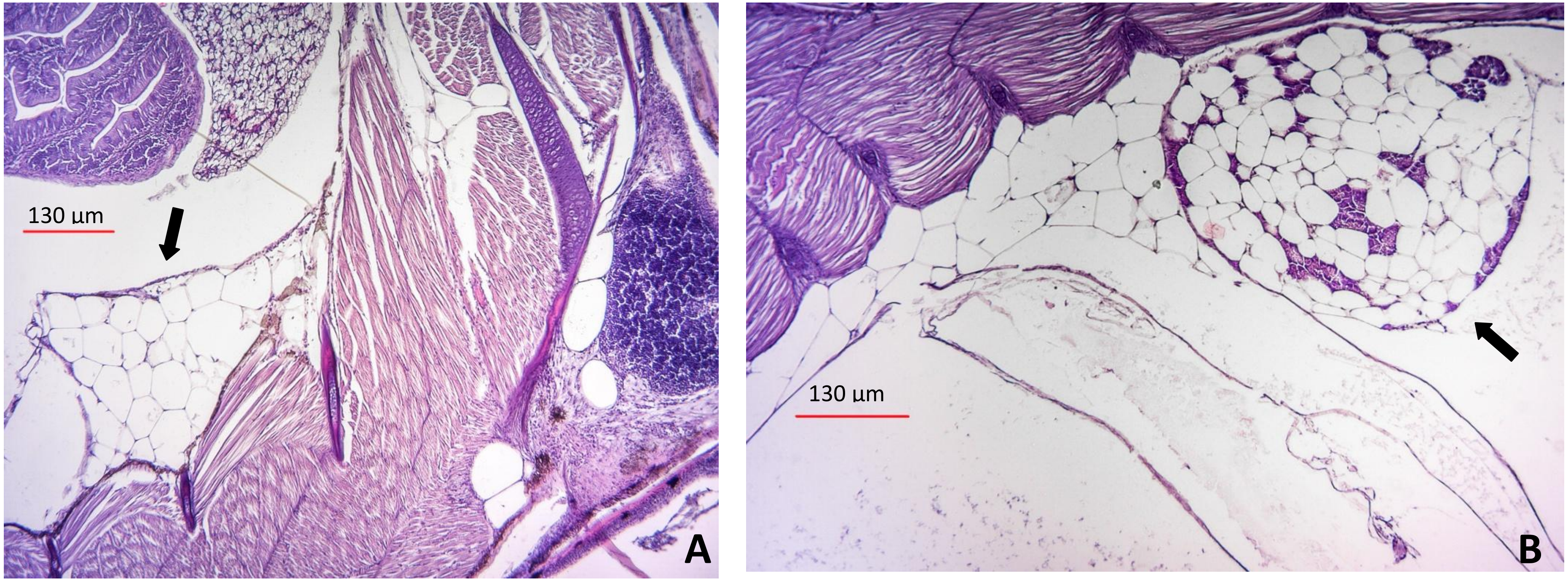

S1 Fig. Histological samples of the abdominal cavity. Adipocytes (black arrows), sometimes with pancreatic tissue associated, conform visceral mass surrounding intestine, liver (A) and swim bladder (B) in mosquitofish juveniles. Magnification x100.
