# Supplementary material for: Ecologically relevant biomarkers reveal that chronic effects of nitrate depend on sex and life stage in the invasive fish Gambusia holbrooki
Source: PLoS One. 2019 Jan 28;14(1):e0211389. doi: 10.1371/journal.pone.0211389 (PMC6349331; doi:10.1371/journal.pone.0211389)
Supplement: S2 Fig — (PDF) [file pone.0211389.s011.pdf]

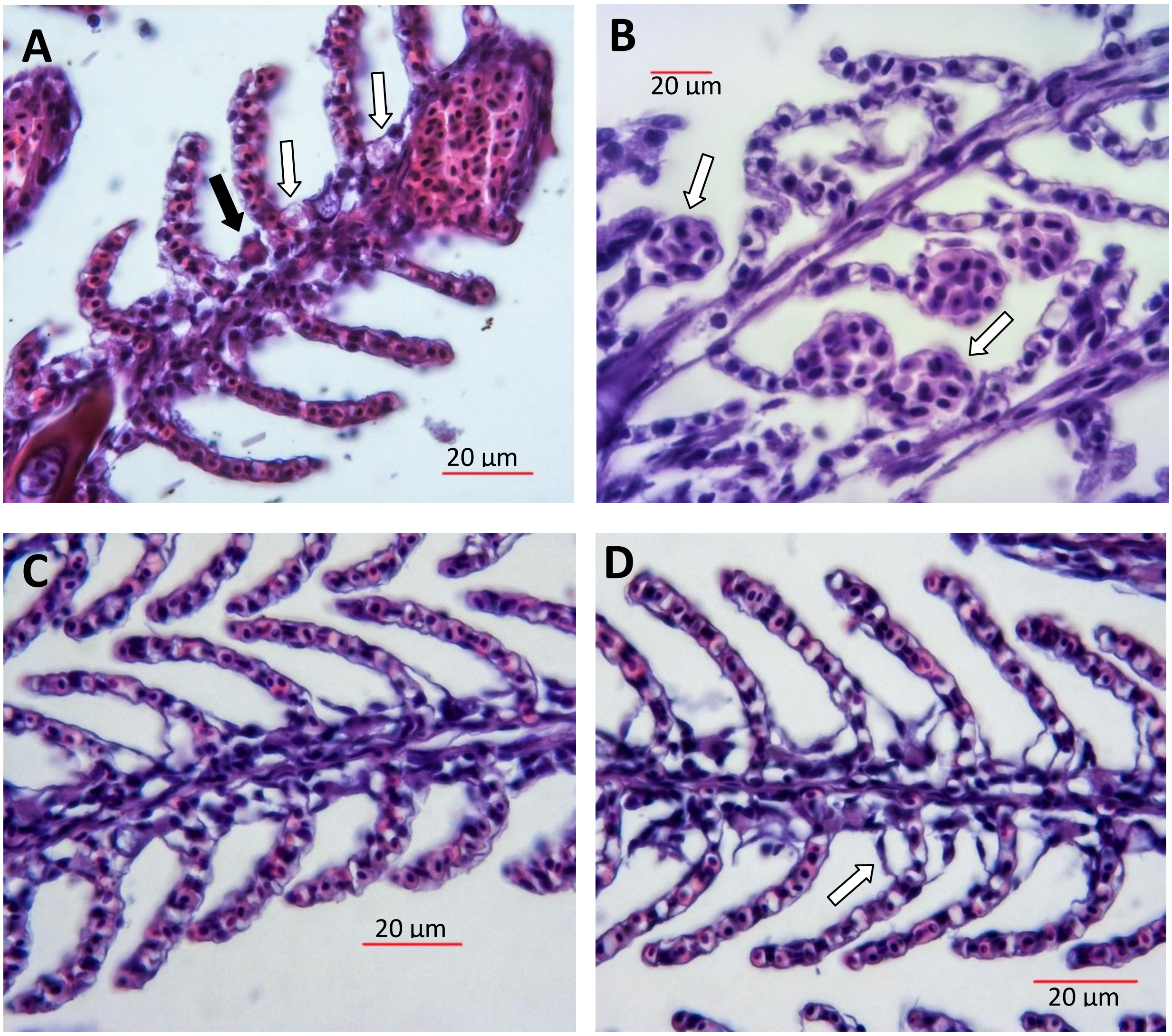

S2 Fig. Histological samples of the gill tissue examined. No differences were found between control and nitrate exposed mosquitofish individuals at 50 and 250 mg/l. A) Normal gill structure, with slight congestion in the blood vessels. Black arrow: mucous cell in the gill epithelium. White arrow: chloride cells at the base of the secondary lamella. No hyperplasia or changes of both cell types were found in nitrate treatments compared to control. B) Small telangiectasias (white arrows) occasionally appeared in few individuals. These small dilations of blood vessels are likely attributable to the euthanasia methods. C and D) Normal aspect of gill filament with secondary lamellae in females. Slight epithelial lifting at the base (white arrow) can be occasionally found in fish at different exposure levels. These changes are considered fixation artefacts. Magnification x400.
