# Supplementary material for: Ecologically relevant biomarkers reveal that chronic effects of nitrate depend on sex and life stage in the invasive fish Gambusia holbrooki
Source: PLoS One. 2019 Jan 28;14(1):e0211389. doi: 10.1371/journal.pone.0211389 (PMC6349331; doi:10.1371/journal.pone.0211389)
Supplement: S3 Fig — (PDF) [file pone.0211389.s012.pdf]

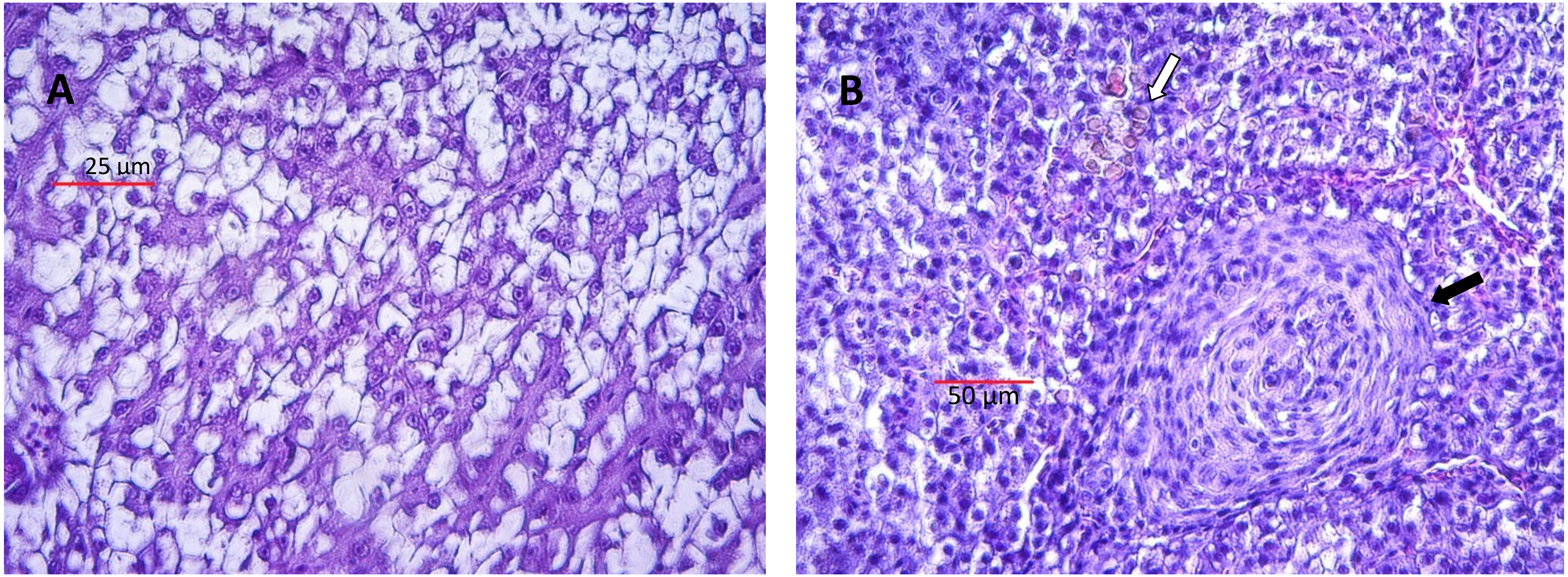

S3 Fig. Histological samples of the hepatic tissue. A) Normal layout of hepatocytes with white areas within the cytoplasm. B) Occasional presence of granulomas (black arrow) in few individuals of adult mosquitofish. We could not ascertain their origin, possibly after Microsporidia or parasite infection. Macrophage aggregates (white arrow) in the vicinity of the granuloma. These aggregates, which tend to increase in number and size in different organs as fish grows, were also present in males and especially females. Magnification x400, x200.
