# Supplementary material for: Ecologically relevant biomarkers reveal that chronic effects of nitrate depend on sex and life stage in the invasive fish Gambusia holbrooki
Source: PLoS One. 2019 Jan 28;14(1):e0211389. doi: 10.1371/journal.pone.0211389 (PMC6349331; doi:10.1371/journal.pone.0211389)

S7 Table. Spearman rank correlation coefficients examining the associations amongst all biomarkers in males. The lower panel shows the graphical values of bivariate correlations with a locally weighted scatterplot smoothing (*LOWESS*) curve. In the upper panel the size of the values are set according to the absolute value of Spearman's rho (ρ). P-values are indicated in red by: ** P<0.001, * P<0.01; ● P<0.05

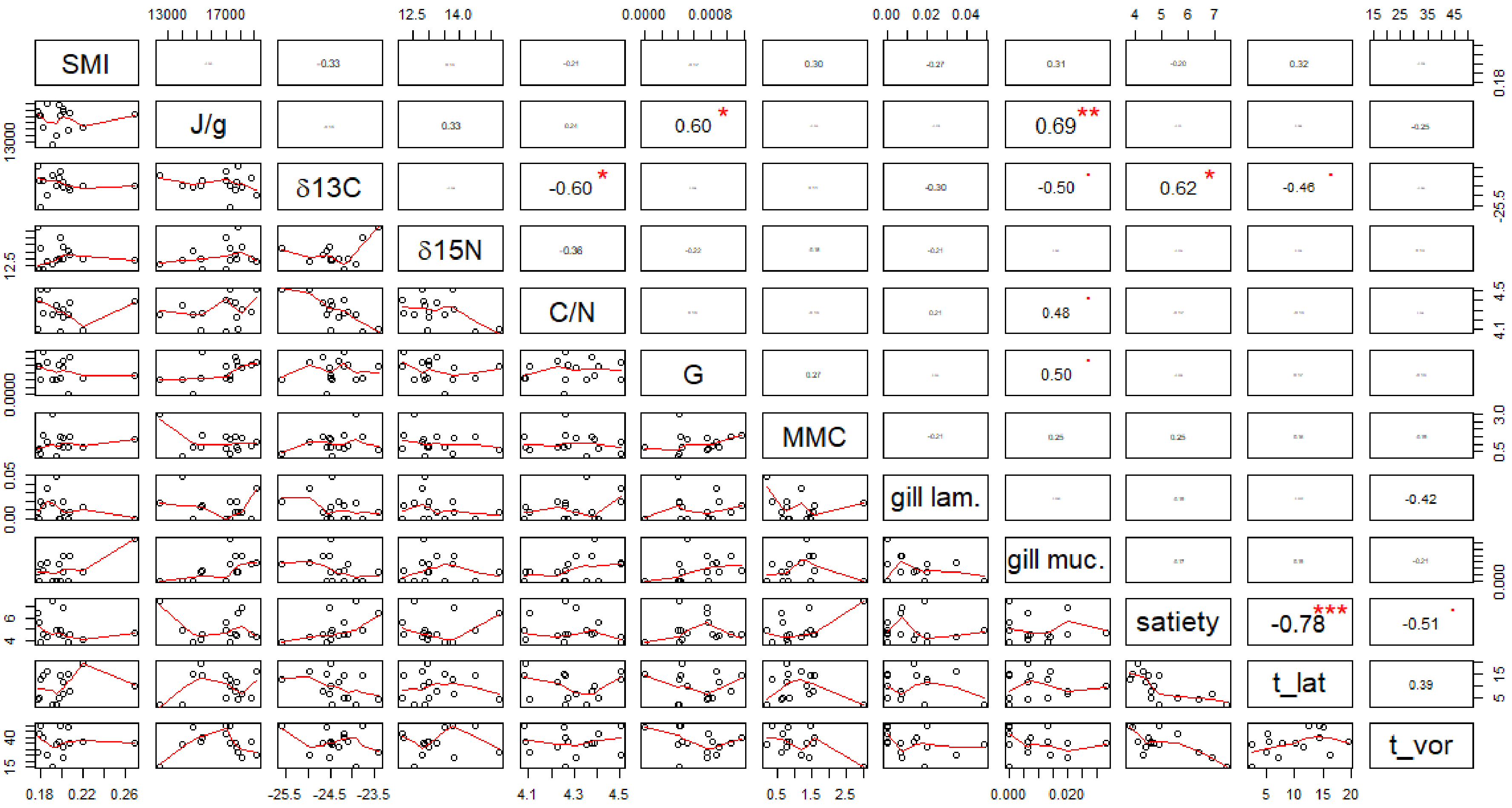

Supplement: S7 Table — (PDF) [file pone.0211389.s007.pdf]
